# Identification and validation of superior reference gene for gene expression normalization via RT-qPCR in staminate and pistillate flowers of *Jatropha curcas* – A biodiesel plant

**DOI:** 10.1371/journal.pone.0172460

**Published:** 2017-02-24

**Authors:** Palaniyandi Karuppaiya, Xiao-Xue Yan, Wang Liao, Jun Wu, Fang Chen, Lin Tang

**Affiliations:** 1 Department of Botany, Key Laboratory of Bio-Resources and Eco-Environment, Ministry of Education, Sichuan University, Chengdu, China; 2 College of Life Science, Sichuan University, Chengdu, China; Kumamoto University, JAPAN

## Abstract

Physic nut (*Jatropha curcas* L) seed oil is a natural resource for the alternative production of fossil fuel. Seed oil production is mainly depended on seed yield, which was restricted by the low ratio of staminate flowers to pistillate flowers. Further, the mechanism of physic nut flower sex differentiation has not been fully understood yet. Quantitative Real Time—Polymerase Chain Reaction is a reliable and widely used technique to quantify the gene expression pattern in biological samples. However, for accuracy of qRT-PCR, appropriate reference gene is highly desirable to quantify the target gene level. Hence, the present study was aimed to identify the stable reference genes in staminate and pistillate flowers of *J*. *curcas*. In this study, 10 candidate reference genes were selected and evaluated for their expression stability in staminate and pistillate flowers, and their stability was validated by five different algorithms (ΔCt, BestKeeper, NormFinder, GeNorm and RefFinder). Resulting, *TUB* and *EF* found to be the two most stably expressed reference for staminate flower; while *GAPDH1* and *EF* found to be the most stably expressed reference gene for pistillate flowers. Finally, RT-qPCR assays of target gene *AGAMOUS* using the identified most stable reference genes confirmed the reliability of selected reference genes in different stages of flower development. *AGAMOUS* gene expression levels at different stages were further proved by gene copy number analysis. Therefore, the present study provides guidance for selecting appropriate reference genes for analyzing the expression pattern of floral developmental genes in staminate and pistillate flowers of *J*. *curcas*.

## Introduction

Physic nut (*Jatropha curcas* L) is a multipurpose, poisonous, drought resistant, semi-evergreen shrub/small tree belonging to the family Euphorbiaceae and it has spread beyond its center of origin to tropical and subtropical regions because of its simple propagation, rapid growth, drought resistance and wide adaptability to variety of soils and different climatic conditions [1,2]. In the last decade, physic nut seed oil has gained much interest worldwide as a potential renewable natural resource for the production of bio-diesel and bio-jet fuel to replace fossil fuels [3] The viscous oil obtained from seeds has medicinal value, that has long been used as a source of lamp oil and used for manufacturing candles, soap, paraffin and cosmetic industry [4,5]. Additionally, the residual seed cake after extraction of oil can be used as biomass to power electricity plants, fertilizer, animal fodder and it can also be used as feed in digesters and gasifiers to produce biogas [6].

Flowers are reproductive organs that are widely studied for molecular breeding, sex determination and improved production of seeds in agronomically important plants. In physic nut, the productivity factor depends on ratio between staminate and pistillate flowers (1/10–1/30) [7]. A study of physic nut inflorescence has shown that the ratio of staminate flower to pistillate flower is low and different proportions occur in different seasons in most of the available germplasm [8]. In the recent years, forward and reverse genomics approaches have been employed to improve physic nut growth, high seed yield with high oil content and more staminate flowers [3]. For sex determination and to improve the number of staminate flowers of physic nut, differential expression of relative genes in staminate and pistillate flowers should be understood. Although, a report showed selection of best candidate reference genes in different tissues of physic nut at different environmental conditions [9], but there is no detailed studies on selection of potential candidate reference genes in staminate and pistillate flowers of physic nut.

Quantifying gene expression pattern is an important factor for unraveling the function of important pathway genes in biological samples. In connection to this, Real-time quantitative PCR (RT-qPCR) is widely used as the most accurate and most reliable technique that can detect and measure small number of mRNA copies due to its high sensitivity, accuracy, specificity and high throughput [10]. However, the accuracy of qPCR is influenced by a number of factors, such as RNA stability, quantity, purity, enzymatic efficiency in cDNA synthesis and PCR amplification [11]. An improper reference gene can lead to misinterpretation of expression data and thus generate incorrect results [12]. Thus, to avoid the influence of these factors, it is a pre-requisite to select ideal reference gene(s) to normalize RT-qPCR analysis and its expression is presumed stable in control and experimental conditions [13,14]. It is therefore pivotal to identify the best potential reference genes for the experimental system under study. Based on following background information, the present study is carried out with an aim to choose these steadily expressed potential genes in the transcriptome data base for the selection of reliable reference gene expression in staminate and pistillate flowers of physic nut.

## Materials and methods

### Plant materials

The flowers were collected from physic nut plants grown at Jinhe town (26°56′N, 101°68′E) located in Yanyuan County, Sichuan Province, China (Permission provided by Yanyuan county Government, China) and stem cutting grown at Green house, College of Life Sciences, Sichuan University, China, collected from Haikou city, Hainan Province (Permission provided by Haikou city Government, China) (S1 Fig). Inflorescences were collected twice from in between April to June and July to September in wild types and stem cuttings. One inflorescence was collected from one *J*. *curcas* plant and totally 20 inflorescence samples were randomly collected each time from *J*. *curcas*. Two to three staminate and pistillate flowers were collected from one inflorescence separately in earlier, middle and later according to the flower developmental stages described by Wu et al [15] (S2 Fig). The flowers of earlier stages were separated from inflorescence under the stereo microscope (Olympus, SZ2). The collected flower samples were immediately frozen in liquid nitrogen and stored at −80°C for further experimental analysis.

### RNA isolation and cDNA synthesis

Total RNA was isolated using TaKaRa MiniBEST Plant RNA Extraction reagents according to the manufacturer’s instructions (Takara Bio. Inc., Dalian, China). The quantity and quality of total RNA samples were assessed by measuring the absorbance ratio at 260/280 and 260/230 nm using a spectrophotometer (Nanovue, Healthcare Bio-Sciences AB, Sweden). RNA samples with absorbance ratio between 1.9 and 2.2 were retained for further analysis. The total RNA integrity was evaluated on a 2% agarose gel (S3 Fig). The first cDNA strand was synthesized using an aliquot of 2.0 μg of total RNA in a 50 μl reaction using reverse transcription system kit (PrimeScript^™^ RT Reagent Kit, Takara Bio. Inc., Dalian, China), following the manufacturer’s protocol. All of the cDNA samples were diluted at 1:10 with RNase-free water and stored at −80°C.

### Selection of candidate reference genes and design of RT-qPCR primer

The cDNA sequences of these endogenous candidate reference genes (*ACTIN*, *EF*, *SLEEPER*, *GAPDH1*, *GAPDH2*, *TUB*, *UBI*, *DSK2A*, *CYC*, and *PLA*) were obtained from the NCBI GeneBank database. RT-qPCR primers were designed using Primer Premier 6 software with melting temperature between 59 to 61°C, primer length between 20 to 23 nucleotides, and PCR amplicon length within 103 to 165 base pair (Table 1).

**Table 1:** Candidate reference genes and its primer sequences.

Gene Name	Gene symbol	Gene Accession No	Primer sequences	Amplicon length
**Actin**	*ACTIN*	XM_012232381.1	F:5'-TCTCGACTACGAGCAGGAGC-3' R:5'-CGGAATCGCTCAGCACCAAT-3'	108bp
**Elongation factor 1-alpha**	*EF*	XM_012226913.1	F:5'-GTCTGTTGAGATGCACCATGAAG-3' R:5'-TAGAGGCAACAAAACCACGTTTC-3'	108bp
**Zinc finger BED domain-containing protein DAYSLEEPER-like**	*SLEEPER*	XM_012217757.1	F:5'-CGTTGCACATAGTGGAACATCC-3' R:5'-AAATTGACACGCATTCCCCTTG-3'	103bp
**Glyceraldehyde 3-phosphate dehydrogenase**	*GAPDH1*	XM_012231954.1	F:5'-TCTGCTGATGCACCAATGTTTG-3' R:5'-ACCTTAGCAAGAGGAGCAAGAC-3'	116bp
**NADP-dependent-glyceraldehyde 3-phosphate dehydrogenase**	*GAPDH2*	XM_012221054.1	F:5'-GTCTGGGGATTTGGTGTCGTAT-3' R:5'-GGATAGTTGAAGGGTGGGATAGC-3'	165bp
**Tubulin beta-5 chain**	*TUB*	XM_012218098.1	F:5'-TTCTGGAATGGGAACTCTGTTGA-3' R:5'-TTGATGTACAGAAAGGGTGGCAT-3'	142bp
**Ubiquitin-conjugating enzyme E2 32**	*UBI*	XM_012228705.1	F:5'-TCAAGTGCTAACTCTGAGTCCAC-3' R:5'-CAGCAGTATTTGACCTTGTGGTG-3'	127bp
**Ubiquitin domain-containing protein DSK2a**	*DSK2A*	XM_012210059.1	F:5'-AAAGGTGTCGGCTTTAGGGAAT-3' R:5'-TCCACCATGGATGACTGACAAG-3'	104bp
**Peptidyl-prolyl cis-trans isomerase CYP21-4**	*CYC*	XM_012227533.1	F:5'- AGTGATAGTCTTGGCGCTGC -3' R:5'- GATCTGGGATAGGTGCAGTTGT -3'	161bp
**Phospholipase A I**	*PLA*	XM_012235990.1	F:5'-CGTTGAGCAAAGCTGGTTCC -3' R:5'- CACAACCATCGCCAAATCCC-3'	103bp

### RT-qPCR analysis

RT-qPCR reactions were carried out with a CFX connected real-time Bio-Rad system using SYBR^®^ green Premix Ex Taq^™^ II (Takara Bio. Inc., Dalian, China). Each 25 μl reaction volume contained 2 μl cDNA, 12.5 μl SYBR^®^ Premix Ex Taq^™^ II, 8.5 μl dH_2_O, and 1 μl each primer. The reaction conditions included an initial denaturation step of 95°C for 30 s, followed by 40 cycles of 95°C/5 s and 60°C/30 s. The dissociation curve was obtained by heating the amplicon from 65 to 95°C. Each RT-qPCR reactions were performed in three technical replicate and three biological replicate. A non-template control was also included for each gene. The final quantification cycle (Cq) values were the means of nine values (biological triplicate, each in technical triplicate).

### Analysis of expression stability determination using different statistical algorithms

To obtain high accuracy of stability ranking from the C_q_ values of each candidate reference gene, four different statistical algorithms such as ΔC_t_ [16], geNorm (version 3.5) [17], BestKeeper (version 1.0) [18], and NormFinder (version 0.953) [19] were used to evaluate expression stability of reference genes. The RT-qPCR data obtained from the Bio-Rad CFX connected Real-time system were exported into an Excel datasheet. The raw Cq values (S1 Table) were used directly for stability calculations in BestKeeper analysis and ΔC_t_ method. Then the raw Cq values were converted into relative quantities and imported into the geNorm and Norm-Finder analysis using the formula Q = E^-ΔCq^, in which E = amplification efficiency, ΔCq = the corresponding Cq value -minimum Cq. Genes with the lowest standard deviation (SD) and coefficient of variance (CV) values are considered as the most stable reference genes. In geNorm, the reference gene expression stability measurement (M) value was calculated as the level of pairwise variation for each reference gene with all other control genes and as the standard deviation (SD) of the logarithmically transformed expression ratios [17]. The RefFinder (http://www.leonxie.com/referencegene.php), a web-based comprehensive tool integrating the data obtained from ΔC_t_ method, Genorm, BestKeeper and NormFinder to calculate the recommended comprehensive ranking order [20]. All the software packages were used according to the manufacturer’s instructions. All other multiple comparisons were performed with GrapPad Prism 7 statistical software.

### Validation of reference gene by expression analysis of *AGAMOUS* gene

Physic nut *AGAMOUS* gene is a floral organ identity gene in C class of ABC model which specifies stamen and carpel development [21]. Expression of floral organ identifying gene, *AGAMOUS*, was used as a target gene to demonstrate the usefulness of the selected candidate reference genes in RT-qPCR. For the validation of selected reference gene, the expression level of *AGAMOUS* gene was analyzed using the most stable reference genes and the most varying reference genes in staminate and pistillate flower samples, which was calculated by 2^-ΔΔCq^ method. These experiments were performed in triplicate for each sample.

### *AGAMOUS* gene isolation and calculation of plasmid copy number

*AGAMOUS* gene was isolated using the following PCR primers: Forward primer: 5’-ATCAGCAACAAGCTGCCAAG-3’ and Reverse primer: 5’-CTGCTCTCCAAGCCCCTAAG-3’. The PCR product was cloned into the pUC57 vector. Plasmid DNA concentration was estimated by UV spectrophotometer at 260 nm. Plasmid copy number was calculated as described in detail previously [22] with minor modification using the following formula:

$$\text{Number of copies }\left(\text{copies }/\;\mu \text{L}\right)=\frac{6.02\;\times \;10^{23}\;\left(\text{copies }/\text{ mol}\right)\;\times \text{ plasmid concentrations }\left(\text{g }/\;\mu \text{L}\right)\;}{\left(\text{number of base pairs }\times \;660\text{ g }/\text{ mol}\right)}$$

### Establishment of standard curve for absolute quantification

The purified plasmid was diluted with sterile deionized water to obtain a standard series from 1.49 × 10^3^ to 1.49 × 10^7^ copies / μL. Quantitative RT-PCR was performed using 25 ng / μL DNA with three replicates. The standard curve was established (S4 Fig) as described in details previously [23].

## Results

### Specificity and efficiency of RT-qPCR amplification of the candidate reference genes

**Fig 1:**
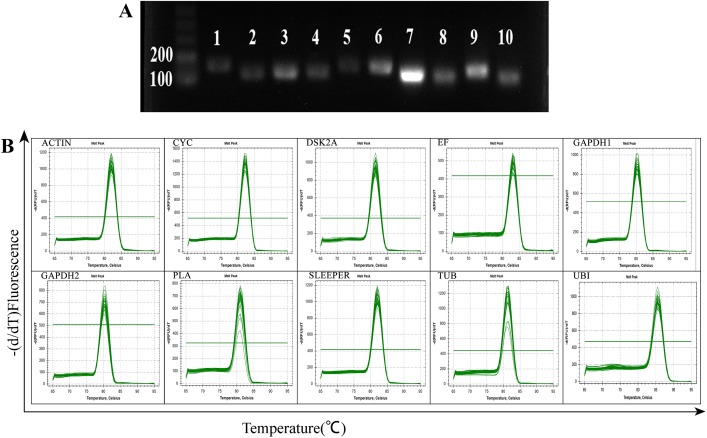
**A. Primer specificity and amplicon size.** Agarose gel electrophoresis (2.0%) indicates amplification of a single PCR product of the expected size for 10 genes (Line 1–10: *ACTIN*, *EF*, *SLEEPER*, *GAPDH1*, *GAPDH2*, *TUB*, *UBI*, *DSK2A*, *CYC*, and *PLA***). B. Melting curve analysis:** Melting curves of 10 genes show single peaks.

Potentially useful endogenous reference genes were selected based on previous studies, which are generally used for normalization and also routinely used as control for RT-PCR or blotting techniques. We selected 10 candidate reference genes (*ACTIN*, *EF*, *SLEEPER*, *GAPDH1*, *GAPDH2*, *TUB*, *UBI*, *DSK2A*, *CYC*, and *PLA*) to normalize the gene expression levels in physic nut inflorescence containing staminate and pistillate flowers at three developmental stage using RT-qPCR. Specific primer pairs were designed for each candidate reference gene, with the amplicon lengths ranging from 103 bp to 165 bp. A single DNA band at the correct molecular weight for each product in agarose gel electrophoresis indicating good specificity of all the primer pairs used in RT-qPCR (Fig 1A). The melting curves for the amplified products of all 10 candidate genes showed a single peak corresponding to a specific melting temperature (Fig 1B). PCR amplification efficiencies of each candidate reference gene were calculated from standard curves with significant linear relationship (R^2^ > 0.99); amplification efficiencies were in between 92.4% to 106.2% (S2 Table).

### Expression profile of reference genes as quantification cycle (Cq)

**Fig 2:**
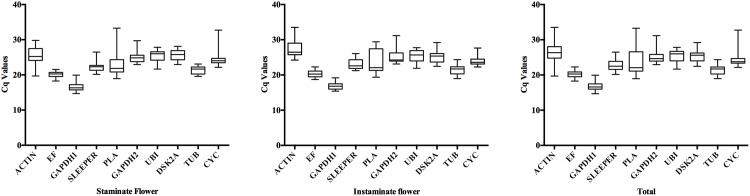
The quantification cycle (Cq) values of the candidate reference genes in staminate, pistillate and total samples. Lines across the Box-plot graph of Cq values represent the median values. Lower and upper boxes show the 25^th^ percentile to the 75^th^ percentile. The whisker caps represent the maximum and minimum values.

To provide an overview of the transcript levels, the Cq values of the 10 candidate reference genes were obtained by RT-qPCR using both staminate and pistillate flower samples at three developmental stages. The Cq values of all 10 candidate genes were shown in the box-plot chart (Fig 2). Mean Cq values of candidate genes of staminate, pistillate and total ranged from 16.50 (*GAPDH1*) to 25.57 (*DSK2A*); 16.96 (*GAPDH1*) to 27.63 (*ACTIN*); 16.73 (*GAPDH1*) to 26.60 (*ACTIN*). The Cq values of all the tested samples were between 16.50 to 27.63. Among the 10 candidate reference genes, *GAPDH1* had the highest accumulation of the transcript followed by *EF* in both staminate and pistillate flowers of *J*. *curcus*, while *DSK2A* and *ACTIN* had the lowest accumulation of transcript levels, and the remaining genes had intermediate transcript expression levels. The coefficients of variation (CV) of ten reference genes were 4.82% (*EF*), 5.81% (*TUB*), 6.85% (*GAPDH1*), 6.69% (*DSK2A*), 7.01% (*SLEEPER*), 7.36% (*UBI*), 7.87% (*GAPDH2*), 8.75% (*CYC*) and 10.98% (*ACTIN*) (lower values of CV represent lower variability).

### Ranking and determination of the optimal reference genes

#### 1. Analysis using geNorm software

**Fig 3:**
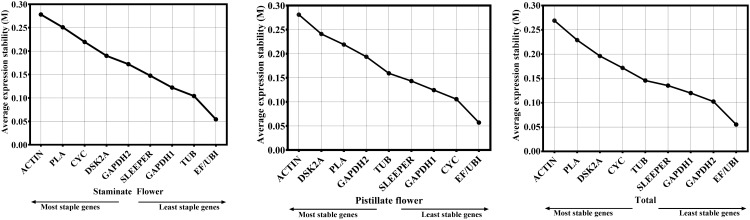
Gene expression stability values (M) and ranking of 10 reference genes as assayed by GeNorm in staminate, pistillate and total samples. The least stable genes are on the left and the most stable genes on the right.

The geNorm software is a visual basic application to determine the most stable reference (housekeeping) genes. Ranking of each gene was produced based on their expression stability. Based on geNorm analysis, *EF* and *UBI* were considered as top most stable reference genes followed by TUB as second rank for their stability in staminate flower samples. In pistillate flowers, *EF* and *UBI* were ranked as first for their stability followed by *CYC* as second most stable reference gene. Whereas, *ACTIN* and *PLA; Actin* and *DSK2A* were least expressed, and hence, inconsistent genes in staminate and pistillate flower samples respectively (Fig 3).

**Fig 4:**
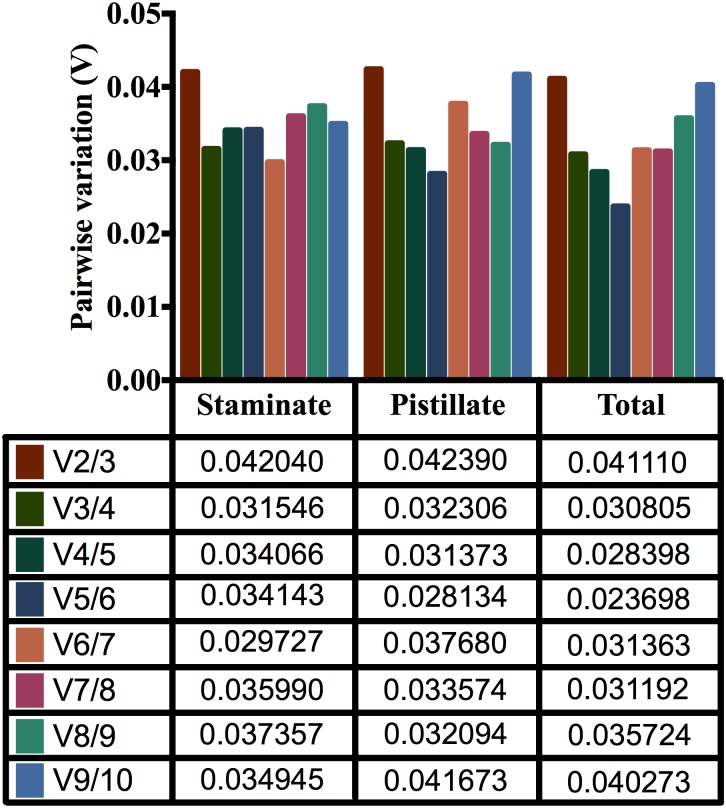
Pairwise variation (V) of the candidate reference genes calculated by geNorm in staminate, pistillate and total samples. Vn/Vn+1 value were used for decision of the optimal number of reference genes.

GeNorm also computes a pairwise variation (V_n_/V_n+1_) analysis between normalization factors NFn and NFn+1 to determine the benefit of adding extra reference genes for the normalization. Adding another gene is unnecessary when small variation exists between V_n_/_n+1_ and V_n+1_/V_n+2_. A threshold value of 0.15 may be used to determine the necessity of the addition of more reference genes [17,24]. In our analysis, data shows V_2+3_ values of staminate and pistillate flower were lower than 0.15, indicating that two reference genes were suitable for gene normalization (Fig 4). Therefore, pairwise variation analysis suggested that two reference genes could be useful for gene normalization of staminate and pistillate flower samples.

#### 2. NormFinder software analysis

The NormFinder software program generates a stability value for evaluating expression variation when using reference genes for normalization. According to the NormFinder software, the gene that has the lowest stability value (SV) is the most stable reference gene for RT-qPCR. The stability values obtained from all 10 reference genes were listed in Table 2. NormFinder analysis results revealed that *TUB* (0.53), *EF* (0.99) and *SLEEPER* (1.00) were the top three stable reference genes in staminate flower samples, while *GADPH1* (0.46), *SLEEPER* (0.54) and *EF* (0.69) were identified as top three stable reference genes in pistillate flower. TUB was top most stable gene in staminate flower but in pistillate flower, it exhibited low stability and ranked as sixth position. Similarly, *GADPH1* was ranked as top most stable gene in pistillate flower, but in staminate flower samples, it ranked as fourth position. *PLA* and *ACTIN* exhibited the most unstable value and identified as low stable gene in both staminate and pistillate flower samples.

**Table 2:** Expression stability analysis of reference genes assayed by NormFinder software.

Ranking order	Staminate		Pistillate		Total	
	Genes Name	Stability Value	Genes Name	Stability Value	Genes Name	Stability Value
1	*TUB*	0.53	*GAPDH1*	0.46	*SLEEPER*	0.80
2	*EF*	0.99	*SLEEPER*	0.54	*EF*	0.84
3	*SLEEPER*	1.00	*EF*	0.69	*GAPDH1*	0.88
4	*GAPDH1*	1.17	*CYC*	1.18	*TUB*	1.15
5	*GAPDH2*	1.20	*GAPDH2*	1.43	*GAPDH2*	1.31
6	*DSK2A*	1.23	*TUB*	1.55	*DSK2A*	1.63
7	*CYC*	2.29	*DSK2A*	1.92	*CYC*	1.90
8	*UBI*	2.55	*UBI*	2.20	*UBI*	2.36
9	*ACTIN*	3.02	*ACTIN*	2.83	*ACTIN*	3.08
10	*PLA*	4.00	*PLA*	3.17	*PLA*	3.58

#### 3. Analysis using BestKeeper software

The BestKeeper software evaluates the expression stability of the candidate reference genes based on the following three variables the standard deviation (SD), the coefficient of covariance (CV) and the coefficient of correlation (r) [18]. The most stable reference genes produced lower standard deviation values. The stability values of each reference gene were calculated by BestKeeper and the results are as shown in Table 3. *EF* (0.73), *GAPDH1* (0.85) and *TUB* (0.98) were ranked as top three stable reference genes in staminate flower samples, while *EF* (0.83) was ranked as top first stable reference gene similar staminate flower followed by *SLEEPER* (0.92) and *EF* (1.02) as second and third stable genes for pistillate flowers. *PLA* and *ACTIN* exhibited low stability and ranked as least stable reference genes in staminate and pistillate flower samples respectively. In this analysis, *EF* gene was ranked as most stable reference gene in both staminate and pistillate flowers similar to geNorm analysis. The ranking order obtained in present analysis was found different from the results obtained by NormFinder software analysis.

**Table 3:** Expression stability analysis of reference genes assayed by BestKeeper software.

Ranking order	Staminate		Pistillate		Total	
	Genes Name	Stability Value	Genes Name	Stability Value	Genes Name	Stability Value
1	*EF*	0.73	*EF*	0.83	*EF*	0.77
2	*GAPDH1*	0.85	*GAPDH1*	0.92	*GAPDH1*	0.9
3	*TUB*	0.98	*CYC*	1.02	*TUB*	1.05
4	*SLEEPER*	1.25	*TUB*	1.11	*SLEEPER*	1.23
5	*DSK2A*	1.3	*SLEEPER*	1.19	*DSK2A*	1.36
6	*GAPDH2*	1.37	*DSK2A*	1.39	*CYC*	1.37
7	*UBI*	1.54	*UBI*	1.6	*GAPDH2*	1.55
8	*CYC*	1.71	*GAPDH2*	1.72	*UBI*	1.57
9	*ACTIN*	2.06	*ACTIN*	2.35	*ACTIN*	2.12
10	*PLA*	3.11	*PLA*	3.02	*PLA*	3.06

#### 4. ΔC_t_ method

ΔCt analysis evaluates the expression stability of the candidate reference genes based on the standard deviation. The ranking order results obtained by ΔCt analysis were tabulated in Table 4. Based on ΔCt analysis, *TUB* (2.05), *EF* (2.12) and *GAPDH1* (2.26) were identified as top three stable reference genes in staminate flower samples. On the other hand, *GAPDH1* (1.85), *EF* (1.90) and *SLEEPER* (1.92) were recommended as top three stable reference genes in pistillate flowers. While, *PLA* and *ACTIN* exhibited low stability in both samples hence, it was ranked as the least appropriate reference genes. *TUB* and *GAPDH1* showed high stability in both staminate and pistillate flower similar to NormFinder analysis.

**Table 4:** Expression stability analysis of reference genes assayed by ΔCt method.

Ranking order	Staminate		Pistillate		Total	
	Genes Name	Stability Value	Genes Name	Stability Value	Genes Name	Stability Value
1	*TUB*	2.05	*GAPDH1*	1.85	*EF*	2.05
2	*EF*	2.12	*EF*	1.9	*GAPDH1*	2.1
3	*GAPDH1*	2.26	*SLEEPER*	1.92	*SLEEPER*	2.15
4	*SLEEPER*	2.28	*CYC*	2.1	*TUB*	2.19
5	*DSK2A*	2.28	*TUB*	2.25	*GAPDH2*	2.36
6	*GAPDH2*	2.37	*GAPDH2*	2.29	*DSK2A*	2.4
7	*CYC*	2.99	*DSK2A*	2.43	*CYC*	2.65
8	*UBI*	3.01	*UBI*	2.66	*UBI*	2.85
9	*ACTIN*	3.54	*ACTIN*	3.22	*ACTIN*	3.53
10	*PLA*	4.27	*PLA*	3.48	*PLA*	3.89

#### 5. RefFinder software analysis

Finally, RefFinder tool was used to generate an integrated comprehensive ranking of the most stable candidate reference genes based on different software programs, including ΔC_t_, GeNorm, NormFinder, and BestKeeper. Results obtained in RefFinder analysis were formulated in Table 5. According to the comprehensive ranking analysis of RefFinder, the most stable genes were *EF* and *GAPDH1* for total (staminate and pistillate), whereas *TUB* and *EF* for staminate flower samples, and *GAPDH1* and *EF* for pistillate flower samples. *EF* exhibited stability and ranked as second stable reference gene in both staminate and pistillate flower samples. While, *PLA* and *ACTIN* exhibited low stability in both samples and ranked as the most unstable reference genes in staminate and pistillate flower samples.

**Table 5:** Most stable and least stable reference genes based on RefFinder analysis.

Ranking Order (Better—Good—Average)										
Method	1	2	3	4	5	6	7	8	9	10
**Staminate**										
Delta CT	*TUB*	*EF*	*GAPDH1*	*SLEEPER*	*DSK2A*	*GAPDH2*	*CYC*	*UBI*	*ACTIN*	*PLA*
BestKeeper	*EF*	*GAPDH1*	*TUB*	*SLEEPER*	*DSK2A*	*GAPDH2*	*UBI*	*CYC*	*ACTIN*	*PLA*
Normfinder	*TUB*	*EF*	*SLEEPER*	*GAPDH1*	*GAPDH2*	*DSK2A*	*CYC*	*UBI*	*ACTIN*	*PLA*
Genorm	*EF/UBI*		*TUB*	*GAPDH1*	*SLEEPER*	*GAPDH2*	*DSK2A*	*CYC*	*PLA*	*ACTIN*
Recommended comprehensive ranking	*TUB*	*EF*	*GAPDH1*	*DSK2A*	*SLEEPER*	*GAPDH2*	*UBI*	*CYC*	*ACTIN*	*PLA*
**Pistillate**										
Delta CT	*GAPDH1*	*EF*	*SLEEPER*	*CYC*	*TUB*	*GAPDH2*	*DSK2A*	*UBI*	*ACTIN*	*PLA*
BestKeeper	*EF*	*GAPDH1*	*CYC*	*TUB*	*SLEEPER*	*DSK2A*	*UBI*	*GAPDH2*	*ACTIN*	*PLA*
Normfinder	*GAPDH1*	*SLEEPER*	*EF*	*CYC*	*GAPDH2*	*TUB*	*DSK2A*	*UBI*	*ACTIN*	*PLA*
Genorm	*EF/UBI*		*CYC*	*GAPDH1*	*SLEEPER*	*TUB*	*GAPDH2*	*PLA*	*DSK2A*	*ACTIN*
Recommended comprehensive ranking	*GAPDH1*	*EF*	*SLEEPER*	*CYC*	*TUB*	*DSK2A*	*GAPDH2*	*UBI*	*ACTIN*	*PLA*
**Total**										
Delta CT	*EF*	*GAPDH1*	*SLEEPER*	*TUB*	*GAPDH2*	*DSK2A*	*CYC*	*UBI*	*ACTIN*	*PLA*
BestKeeper	*EF*	*GAPDH1*	*TUB*	*SLEEPER*	*DSK2A*	*CYC*	*GAPDH2*	*UBI*	*ACTIN*	*PLA*
Normfinder	*SLEEPER*	*EF*	*GAPDH1*	*TUB*	*GAPDH2*	*DSK2A*	*CYC*	*UBI*	*ACTIN*	*PLA*
Genorm	*EF/UBI*		*GAPDH2*	*GAPDH1*	*SLEEPER*	*TUB*	*CYC*	*DSK2A*	*PLA*	*ACTIN*
Recommended comprehensive ranking	*EF*	*GAPDH1*	*SLEEPER*	*DSK2A*	*TUB*	*GAPDH2*	*UBI*	*CYC*	*ACTIN*	*PLA*

### Reference gene validation

**Fig 5:**
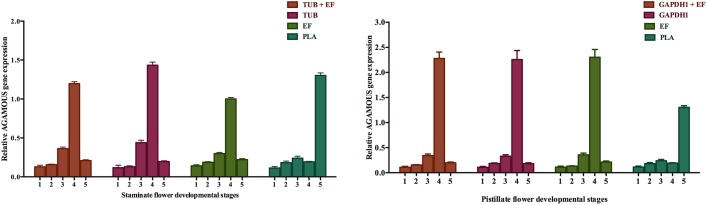
Relative expression of *AGAMOUS* gene using selected reference genes including most stable and least stable reference genes for normalization in staminate and pistillate flowers. The error bars represent standard deviation.

It has been evident that the inappropriate reference genes used for target gene validation can dramatically change the interpretation of the expression pattern [13]. Results of the optimal reference gene study indicated that *TUB* and *EF; GAPDH1* and *EF* were the two top most stable reference genes for RT-qPCR assays for staminate and pistillate flower samples respectively. To confirm the utility of candidate reference genes, the expression of a target gene, *AGAMOUS* in response to floral organ identity gene, which specifies stamen and carpel development was determined. The two top-most stable reference genes *TUB* and *EF; GAPDH1* and *EF* for staminate and pistillate flower samples respectively and the least stable reference gene *PLA* selected from the analysis were used for the validation analysis. When normalized using the two most stable reference genes *TUB* and *EF* as staminate flower internal controls, the relative expression levels of *AGAMOUS* in staminate flower increased in stage 4 and declined in stage 5 (Fig 5). On the other hand, the two most stable genes *GAPDH1* and *EF* were used as pistillate flower internal controls, the expression level of *AGAMOUS* gene increased gradually in stage 1 to stage 4 and then declined at stage 5 (Fig 5). The expression level of *AGAMOUS* gene normalized with *PLA*, which was the least stable reference gene as calculated in both staminate and pistillate flowers, the expression pattern showed fluctuations and failed to achieve a consistent expression pattern in both staminate and pistillate flower samples (Fig 5).

### *AGAMOUS* gene copy numbers determination by quantitative RT-PCR

**Fig 6:**
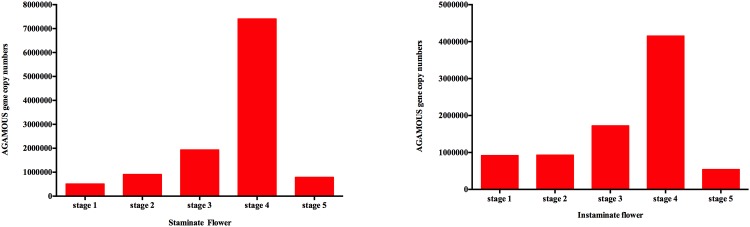
*AGAMOUS* gene copy number. The copy number was calculated by standard curve at different developmental stages for staminate and pistillate flower samples of *Jatropha curcas*.

There is no detailed study on *AGAMOUS* gene expression pattern at different stages of staminate and pistillate flowers of physic nut. Hence, to confirm the selected stable reference genes stability, copy numbers of *AGAMOUS* gene was calculated based on standard curve obtain as described above. Resulting, the *AGAMOUS* gene copy numbers calculated at different stages of staminate and pistillate flower development were shown in Fig 6. *AGAMOUS* gene copy numbers was gradually increasing from stage 1 to stage 3 and then it was high at stage 4 and then it started to decline at stage 5 in both staminate and pistillate flowers (S3 & S4 Tables). Thus, the obtained result was similar to stable reference gene stability analysis data, while, *PLA* the least stable reference gene as internal control was in contrast to the gene copy number analysis data. Therefore, gene copy number analysis further proved that *PLA* was the least stable reference gene.

## Discussion

Gene expression studies could lead to a better understanding of process involved in commercially important bioenergy crop Physic nut flower development. RT-qPCR has become mainstreams of a biological research tools due to its specificity, accuracy, efficiency and reproducibility for understanding the gene expression pattern of target genes which not only provides insights into the complex regulatory networks but also identifies novel genes relevant to key biological processes. To achieve high accuracy, a reference gene should have a relatively stable expression level into distinct biological samples, such as across tissues, developmental stages and experimental conditions. The present work is the first detailed study for the identification of set of control genes for gene normalization of transcript levels in staminate and pistillate flowers of physic nut.

It has been recommended that more than two statistical algorithms should be used for reference gene stability evaluation [25]. Statistical programs, such as geNorm, NormFinder, ΔC_t_ and BestKeeper have been successfully employed to determine the stability of reference gene expression and identify stable reference genes for various plant species. Besides, RefFinder, a comprehensive tool was developed to generate the final overall ranking of tested reference genes based on the geometric mean of every gene calculated by each program. A lower geometric mean of rankings indicates that the gene is more stable, and more narrow error bars indicate that the result is more reliable [25]. In the present study, discrepancies were found in the ranking order among four statistical analytical programs, which might be caused by distinct statistical algorithm procedures. In our study, geNorm analysis ranked *EF*, *UBI* and *TUB* as top most stable genes for staminate flowers, *EF*, *UBI* and *CYC* as best reference genes for pistillate flowers. NormFinder analysis recommended *TUB*, *EF* and *SLEEPER* as top three reference genes for staminate flowers, while *GAPDH1*, *SLEEPER* and *EF* as top three ranked reference genes in pistillate flowers. BestKeeper software generated *EF*, *GAPDH1* and *TUB* for staminate, *EF*, *GAPDH1* and *CYC* for pistillate as top most stable reference genes. ΔC_t_ method computed *TUB*, *EF* and *GAPDH1* for staminate, *GAPDH1*, *EF* and *SLEEPER* for pistillate as the top ranked reference genes. The comprehensive ranking analysis by RefFinder with four statistical programs identified *TUB* and *EF* for staminate, *GAPDH1* and *EF* for pistillate as the top most stable reference genes for RT-qPCR of target gene expression studies. Taken all the results into consideration, all 10 reference genes exhibited differential stability in both flower samples.

Traditionally, a single gene served as reference gene without verification of its stability in different species, tissues and specific environmental conditions. Commonly used reference genes are cellular maintenance genes, which play housekeeping roles in basic cellular components and functions, such as elongation factor 1-α (*EF1-α*), tubulin (*TUB)*, actin (*ACTIN*), glyceraldehyde-3-phosphate dehydrogenase (*GAPDH*), 18S ribosomal RNA (*18s RNA*) and ubiquitin (*UBQ*). However, recent studies indicated that these classical reference genes were used for gene expression studies in different plant species, but its gene expression level among different species, tissues and environmental conditions were varied [26]. In physic nut flower development studies, *TUB1*[7], *18s rRNA* [27], *26s rRNA* and *GAPDH* [28], *ACTIN1* and *ACTIN2* [29] genes were used for gene normalization. However, in this study, *ACTIN* exhibited unstable expression in both staminate and pistillate flower samples. The *TUB* gene, which plays a crucial role in cell structural maintenance, has also been widely used as a reliable reference gene in sweet grass [30] and peach [31]. Similarly, in our study, *TUB* was recommended as most stable reference gene in staminate flowers. However, this gene was ranked as least stably expressed reference gene in all the tested conditions in *Lycoris auria* [32]. *EF* exhibited stable expression in *Glycine max* [33], *Populus euphratica* [34] and *Caragana korshinskii* [35] under various environmental conditions. In this current study, *EF* was ranked as second stable gene in both staminate and pistillate flower samples. *GAPDH1* gene, which encodes a key enzyme involved in the glycolysis and gluconeogenesis [36] is another commonly used reference gene. It has been reported as the most stable reference gene in physic nut over different tissues, developmental stages and environmental conditions [9]. Similarly, in our study *GAPDH1* was the top most stable reference gene in pistillate flowers. The selected stable reference genes in staminate and pistillate flowers are diverse in various studies that evaluated reference genes in different plant species under specific environmental conditions.

In addition, we analyzed the optimal number of reference genes required for accurate normalization using geNorm software. To date, most studies of reference gene selection have reported that when evaluating levels of target gene expression, the results are more pronounced and reliable when two or more reference genes are utilized [37–39]. The pairwise variation studies suggest that a cut off value 0.15 is considered as the ratio, below which there is no additional requirement of any reference gene for normalization [17]. Further, a study indicated that pairwise variation value 0.15 is not an absolute cut off value but rather an ideal value and its use will depend upon the data [40]. However, in our study, the pairwise variation data recommended that two best stable reference genes could be useful for gene normalization in staminate and pistillate flowers of physic nut.

Significant variations of target gene expression levels were found when unstable reference genes were used as the internal control, leading to misinterpretation of experimental results [14,41]. Any gene with a minimal expression level variation in every analyzed sample is considered as best candidate reference gene [32]. To demonstrate the utility of validated reference genes, the expression pattern of *AGAMOUS* gene was examined in staminate and pistillate flowers of physic nut. *AGAMOUS* belongs to the C-class genes in the ABC model of floral organ development, which involve in the regulation of stamen and pistil development [21]. *AGAMOUS* gene expressed remarkably in stage 4 than young stage when *TUB* and *EF*; *GAPDH1* and *EF* in staminate and pistillate flowers respectively. However, when the least stable reference gene *PLA3* was applied, fluctuating expression pattern of *AGAMOUS* gene in both staminate and pistillate flowers. Thus, these results indicate that the appropriate selection of reliable reference genes serves important roles for normalization of target gene expression pattern.

Absolute quantification was employed to determine the copy number of *AGAMOUS* (Gene Bank: XM_012236469) gene cDNA expressed during the different developmental stages of physic nut flower. The absolute quantity analysis of *AGAMOUS* gene expression exhibits *AGAMOUS* gene was expressed at stage 4, which were consistent with the stable reference genes analysis expression pattern, whereas, the least stable reference gene data differed. Hence, this data further confirms that the *TUB* and *EF*; *GAPDH1* and *EF* were the stable reference genes for staminate and pistillate flowers respectively.

## Conclusion

Investigation of ten candidate reference genes in staminate and pistillate flowers of physic nut using five different statistical algorithms indicated that *TUB* and *EF*; *GAPDH1* and *EF* were the two most stable reference genes in staminate and pistillate flowers respectively. On the other hand, *PLA* and *ACTIN* were recommended as the least stable reference gene in both staminate and pistillate flowers. Thus, the stable reference genes identified in this report will enhance accuracy of normalization and quantification of target gene expression with RT-qPCR analysis for staminate and pistillate flower developmental studies in physic nut.

## Supporting information

S1 Fig **A.** Seed cuttings grown at green house **B.** Wild *Jatropha* plants.
(DOCX)

S2 Fig Flower stages. **A.** Staminate flower early stage, **B.** Staminate flower middle stage, **C.** Staminate flower later stage, **D.** Pistillate flower early stage, **E.** Pistillate flower middle stage, **F.** Pistillate flower later stage.
(DOCX)

S3 Fig RNA isolated from different stages of staminate and pistillate flower samples. **1–6: Staminate flower sample from Jinhe town. 1. 1 to 2** staminate flower earlier stage, **3 to 4** staminate flower middle stage, **5 to 6** staminate flower later stage. **7 to 12: Staminate flower stem cutting. 7 to 8:** staminate flower earlier stage; 9 to 10: staminate flower middle stage; 11 to 12: staminate flower later stage; 13 to 18: **Pistillate flower samples from Jinhe town**. **13 to 14** pistillate flower earlier stage, **15 to 16** pistillate flower middle stage, **17 to 18** pistillate flower later stage. 19 to 24: **Pistillate flower samples from stem cutting**. **18 to 19** pistillate flower earlier stage, **20 to 21** pistillate flower middle stage, **22 to 24** pistillate flower later stage.
(DOCX)

S4 Fig Standard curve. (DOCX)

S1 Table Cq values of staminate, pistillate and total samples. (XLSX)

S2 Table PCR amplification efficiency (E), correlation coefficient (R2), Slopes, annealing temperature (Ta) and melting temperature (Tm) of the selected reference genes. (DOCX)

S3 Table Staminate flower AGAMOUS (AG) gene copy number. (XLSX)

S4 Table Pistillate flower AGAMOUS (AG) gene copy number. (XLSX)
