# Correction: Identification and validation of superior reference gene for gene expression normalization via RT-qPCR in staminate and pistillate flowers of *Jatropha curcas* – A biodiesel plant

**DOI:** 10.1371/journal.pone.0177039

**Published:** 2017-05-01

**Authors:** Palaniyandi Karuppaiya, Xiao-Xue Yan, Wang Liao, Jun Wu, Fang Chen, Lin Tang

The author affiliations are incorrect. All authors should be affiliated with: Key Laboratory of Bio-Resources and Eco-Environment, Ministry of Education, College of Life Sciences, Sichuan University, Chengdu, China.
