# Supplementary figures and images for: Identification and validation of superior reference gene for gene expression normalization via RT-qPCR in staminate and pistillate flowers of Jatropha curcas – A biodiesel plant
Source: PLoS One. 2017 Feb 24;12(2):e0172460. doi: 10.1371/journal.pone.0172460 (PMC5325260; doi:10.1371/journal.pone.0172460)

**S1 Figure. A. Seed cuttings grown at green house B. Wild *Jatropha* plants**

**
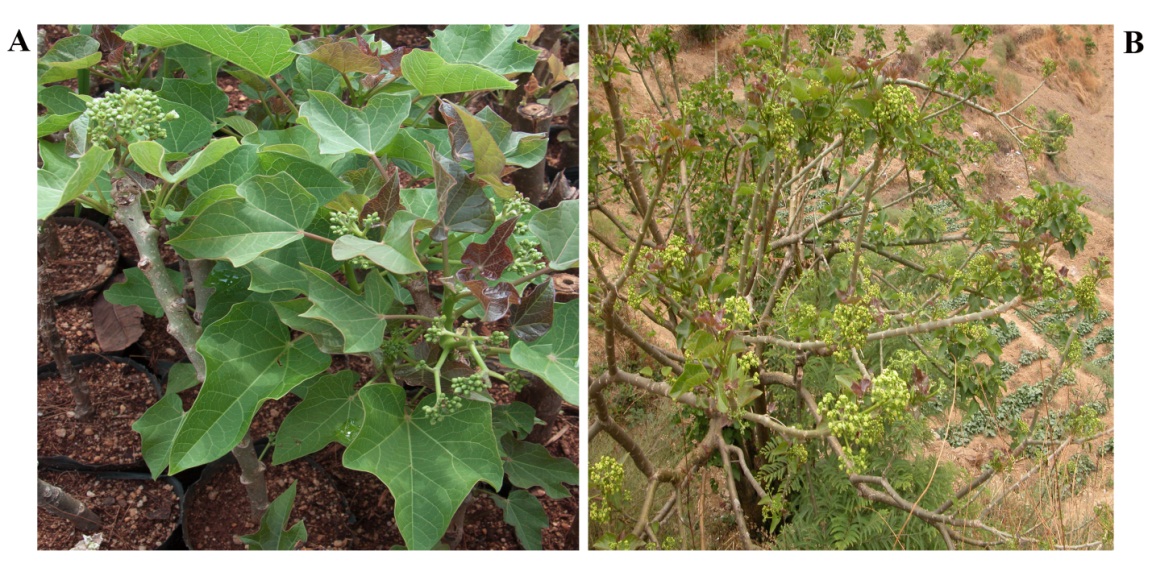
**

Supplement: S1 Fig — A. Seed cuttings grown at green house B. Wild Jatropha plants. (DOCX) [file pone.0172460.s001.docx]

**S4 Figure. Standard Curve.**

**
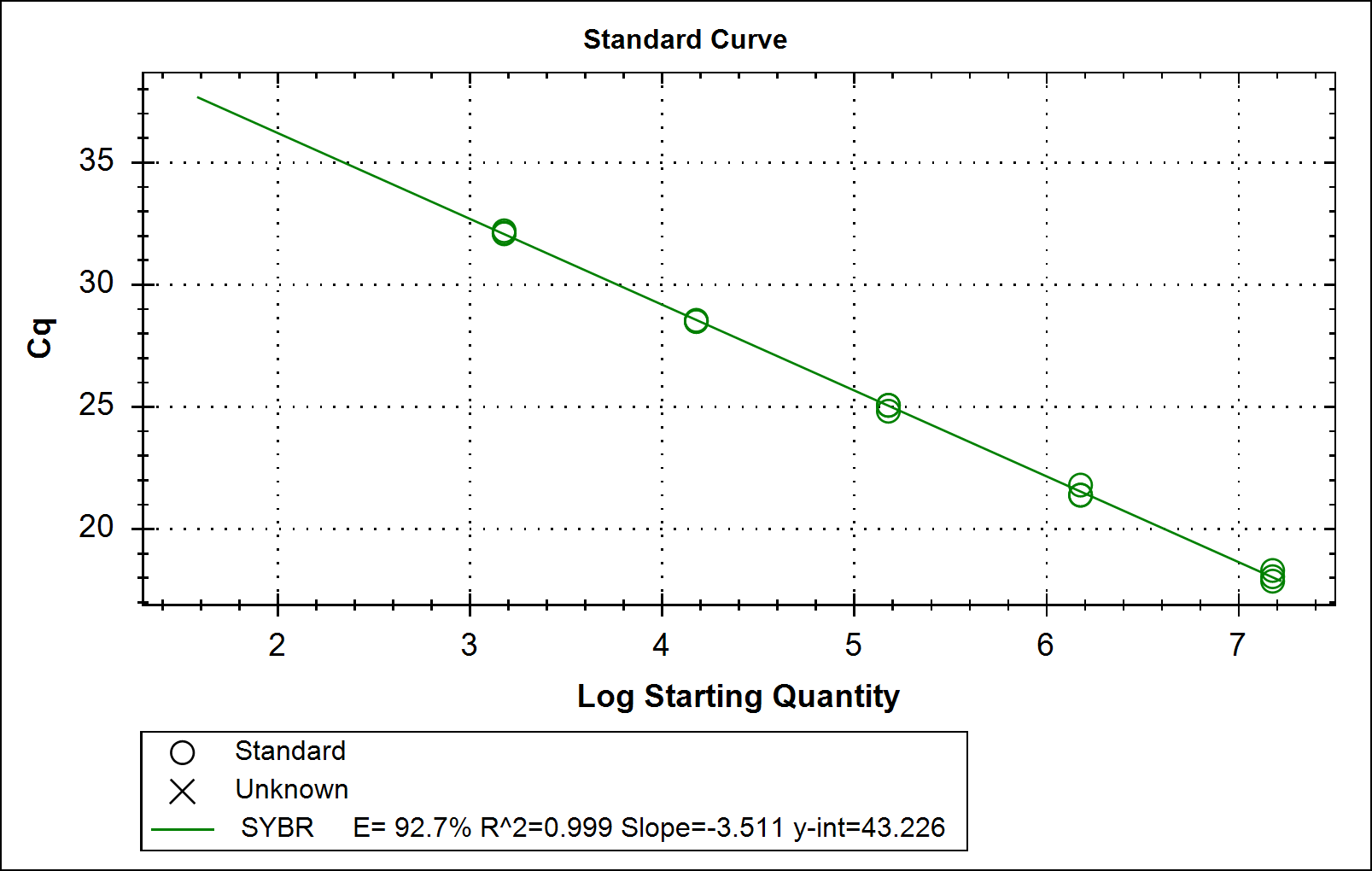
**

Supplement: S4 Fig — (DOCX) [file pone.0172460.s004.docx]
