# Supplementary material for: Identification and validation of superior reference gene for gene expression normalization via RT-qPCR in staminate and pistillate flowers of Jatropha curcas – A biodiesel plant
Source: PLoS One. 2017 Feb 24;12(2):e0172460. doi: 10.1371/journal.pone.0172460 (PMC5325260; doi:10.1371/journal.pone.0172460)
Supplement: S2 Fig — A. Staminate flower early stage, B. Staminate flower middle stage, C. Staminate flower later stage, D. Pistillate flower early stage, E. Pistillate flower middle stage, F. Pistillate flower later stage. (DOCX) [file pone.0172460.s002.docx]

**S2 Figure. Flower stages. A.** Staminate flower early stage, **B.** Staminate flower middle stage, **C.** Staminate flower later stage, **D.** Pistillate flower early stage, **E.** Pistillate flower middle stage, **F.** Pistillate flower later stage**.**

**
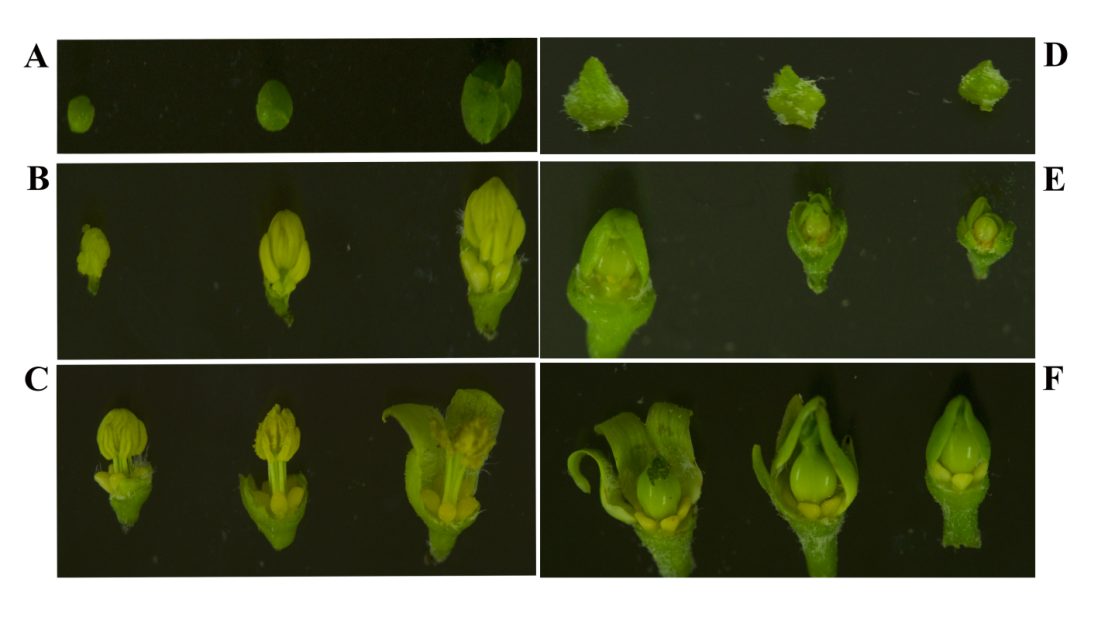
**
