# Supplementary material for: Identification and validation of superior reference gene for gene expression normalization via RT-qPCR in staminate and pistillate flowers of Jatropha curcas – A biodiesel plant
Source: PLoS One. 2017 Feb 24;12(2):e0172460. doi: 10.1371/journal.pone.0172460 (PMC5325260; doi:10.1371/journal.pone.0172460)
Supplement: S3 Fig — 1–6: Staminate flower sample from Jinhe town. 1. 1 to 2 staminate flower earlier stage, 3 to 4 staminate flower middle stage, 5 to 6 staminate flower later stage. 7 to 12: Staminate flower stem cutting. 7 to 8: staminate flower earlier stage; 9 to 10: staminate flower middle stage; 11 to 12: staminate flower later stage; 13 to 18: Pistillate flower samples from Jinhe town. 13 to 14 pistillate flower earlier stage, 15 to 16 pistillate flower middle stage, 17 to 18 pistillate flower later stage. 19 to 24: Pistillate flower samples from stem cutting. 18 to 19 pistillate flower earlier stage, 20 to 21 pistillate flower middle stage, 22 to 24 pistillate flower later stage. (DOCX) [file pone.0172460.s003.docx]

**S3 Figure. RNA isolated from different stages of staminate and pistillate flower samples. 1-6: Staminate flower sample from Jinhe town. 1. 1 to 2** staminate flower earlier stage**, 3 to 4** staminate flower middle stage**, 5 to 6** staminate flower later stage**. 7 to 12: Staminate flower stem cutting. 7 to 8:** staminate flower earlier stage; 9 to 10: staminate flower middle stage; 11 to 12: staminate flower later stage; 13 to 18: **Pistillate flower samples from Jinhe town**. **13 to 14** pistillate flower earlier stage**, 15 to 16** pistillate flower middle stage**, 17 to 18** pistillate flower later stage**.** 19 to 24: **Pistillate flower samples from stem cutting**. **18 to 19** pistillate flower earlier stage**, 20 to 21** pistillate flower middle stage**, 22 to 24** pistillate flower later stage**.**

**
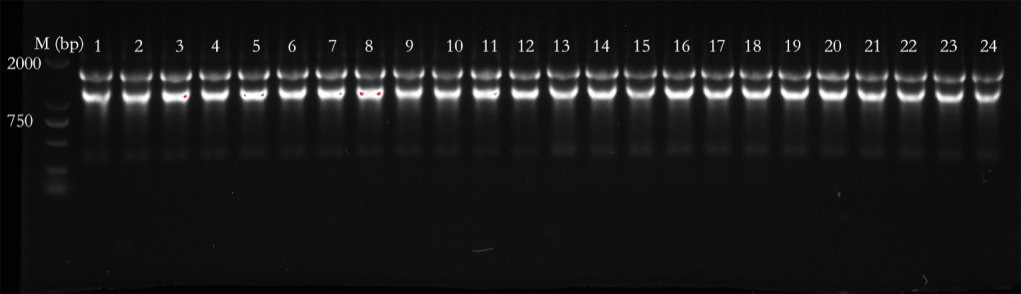
**
