# Supplementary material for: Identification and validation of superior reference gene for gene expression normalization via RT-qPCR in staminate and pistillate flowers of Jatropha curcas – A biodiesel plant
Source: PLoS One. 2017 Feb 24;12(2):e0172460. doi: 10.1371/journal.pone.0172460 (PMC5325260; doi:10.1371/journal.pone.0172460)
Supplement: S2 Table — (DOCX) [file pone.0172460.s006.docx]

**S3 Table.** PCR amplification efficiency (*E*), correlation coefficient (*R^2^*), Slopes, annealing temperature (*Ta*) and melting temperature (*Tm*) of the selected reference genes.

| **Gene symbol** | ***E(%)*** | ***R^2^*** | ***Slope*** | ***Ta* (°C)** | ***Tm* (°C)** |
| --- | --- | --- | --- | --- | --- |
| Actin | 102.1% | 0.991 | -3.274 | 60 | 82.97 |
| EF | 100.8% | 0.984 | -3.718 | 60 | 83 |
| SLEEPER | 93.5% | 0.985 | -3.488 | 60 | 82 |
| GAPDH 1 | 92.4% | 0.992 | -3.519 | 60 | 80.04 |
| GAPDH 2 | 93.7% | 0.982 | -3.484 | 60 | 82.08 |
| PLA | 106.2% | 0.988 | -3.181 | 60 | 81.00 |
| CYC | 98.2% | 0.997 | -3.367 | 60 | 80.93 |
| DSK2A | 95.2% | 0.991 | -3.443 | 60 | 81.47 |
| UBI | 102.3% | 0.996 | -3.267 | 60 | 85.00 |
| TUB | 102.3% | 0.991 | -3.398 | 60 | 77.53 |
